# Differential expression of disulfide reductase enzymes in a free-living platyhelminth (*Dugesia dorotocephala*)

**DOI:** 10.1371/journal.pone.0182499

**Published:** 2017-08-07

**Authors:** Alberto Guevara-Flores, Álvaro Miguel Herrera-Juárez, José de Jesús Martínez-González, Irene Patricia del Arenal Mena, Óscar Flores-Herrera, Juan Luis Rendón

**Affiliations:** Departamento de Bioquímica, Facultad de Medicina, Universidad Nacional Autónoma de México, Apartado, D.F. México, México; Laurentian University, CANADA

## Abstract

A search of the disulfide reductase activities expressed in the adult stage of the free-living platyhelminth *Dugesia dorotocephala* was carried out. Using GSSG or DTNB as substrates, it was possible to obtain a purified fraction containing both GSSG and DTNB reductase activities. Through the purification procedure, both disulfide reductase activities were obtained in the same chromatographic peak. By mass spectrometry analysis of peptide fragments obtained after tryptic digestion of the purified fraction, the presence of glutathione reductase (GR), thioredoxin-glutathione reductase (TGR), and a putative thioredoxin reductase (TrxR) was detected. Using the gold compound auranofin to selectively inhibit the GSSG reductase activity of TGR, it was found that barely 5% of the total GR activity in the *D*. *dorotocephala* extract can be assigned to GR. Such strategy did allow us to determine the kinetic parameters for both GR and TGR. Although It was not possible to discriminate DTNB reductase activity due to TrxR from that of TGR, a chromatofocusing experiment with a *D*. *dorotocephala* extract resulted in the obtention of a minor protein fraction enriched in TrxR, strongly suggesting its presence as a functional protein. Thus, unlike its parasitic counterparts, in the free-living platyhelminth lineage the three disulfide reductases are present as functional proteins, albeit TGR is still the major disulfide reductase involved in the reduction of both Trx and GSSG. This fact suggests the development of TGR in parasitic flatworms was not linked to a parasitic mode of life.

## 1. Introduction

Aerobic organisms are exposed to oxidative stress in the presence of an excess of reactive oxygen species (ROS) generated either under pathological conditions or with variations in the oxygen tension [1–3]. To maintain redox homeostasis, cells have developed both enzymatic and non-enzymatic protective mechanisms [4–8]. Particularly significant in this sense are the glutathione (GSH) and thioredoxin (Trx) antioxidant dependent systems, which have a broad distribution in the living world [9,10]. In their protective role, both GSH and Trx(SH)_2_ act as reducing reagents, leading to its corresponding oxidized form (i.e. GSSG or Trx(S)_2_). To regenerate the reduced state of both compounds, and to maintain a high GSH/GSSG and Trx(SH)_2_/Trx(S)_2_ concentrations ratio, the activity of glutathione reductase (GR) [E. C. 1.8.1.7] and thioredoxin reductase (TrxR) [E.C. 1.8.1.9] enzymes is essential. Both enzymes are NADPH-dependent homodimeric flavoproteins, and its disulfide reductase activity rely upon a conserved thiol/disulfide redox motif [11,12].

Interestingly, while the protein domains and catalytic center of GR have been conserved throughout the evolution, TrxR shows a greater diversity. Thus, in all cases so far reported the 50 to 55 kDa subunits of GR are constituted by three domains (i.e. the NADPH binding domain, the FAD binding domain and the interface domain) and a single dithiol/disulfide catalytically essential redox motif [12]. By contrast, TrxR from prokaryotes and plants exists as a dimer with subunits of about 35 kDa in which the interface domain is absent [13,14]. On the other hand, in animals and protists, two isoforms of TrxR have evolved. In *Plasmodium falciparum* and some insects, the dimeric enzyme is constituted by subunits of about 55 kDa [15,16] with the typical three domain arrangement, and the reductase activity is dependent of two dithiol/disulfide redox motif, one of which is located at the C-terminal end. In mammals, a similar isoform of TrxR is present, differing in the presence of a catalytically essential selenocysteine residue at the C-terminal redox motif, which has substituted for cysteine [17]. Additionally, in these organisms and some other vertebrates, a structurally different third isoform of the enzyme has been described. The enzyme, named as thioredoxin-glutathione reductase (TGR) [E.C. 1.8.1.B1], was initially described from mouse testes [18], and is characterized by the presence of a glutaredoxin (Grx) domain of ~10 kDa fused to the typical animal thioredoxin reductase module (~55 kDa), resulting in subunits of ~65 kDa. The presence of the Grx domain confers to the enzyme the additional catalytic properties to reduce GSSG as well as to perform dithiol/disulfide exchanges, thus making it a true multifunctional protein. In rodents, the enzyme is expressed in significant amounts in mature testes, where it is apparently involved in spermatid maturation [19].

In the parasitic representatives of the flatworms (Platyhelminths), which include flukes (e.g. *Schistosoma mansoni* and *Fasciola hepatica*) and tapeworms (e.g. *Echinococcus granulosus* and *Taenia solium*), the genetic information coding for typical animal GR and TrxR enzymes are absent [20]. Instead, TGR is the only disulfide reductase present, and hence the regeneration of the reduced forms of both glutathione and thioredoxin rely upon the activity of such enzyme. TGR has been characterized from various parasitic flatworms [21–24]. In all cases the enzyme is dependent on a selenocysteine residue for activity, like mammalian TrxR.

Recently, it was reported that in the *Schmidtea mediterranea* (a free-living flatworm representative of the Turbellaria class) genome, nucleotide sequences coding for GR, TGR and TrxR are present [25]. Such observation is in contrast with the situation found in the parasitic flatworms, where the genetic information coding for both GR and TrxR is absent. However, no data about the potential expression of these genes or the activity of such enzymes was given. Considering the different environments in which both set of flatworms thrive, it seems interesting to elucidate the true status of the disulfide reductases in a free-living flatworm. In the present work a search for the potential expression and relative abundance of TrxR, TGR and GR in *Dugesia dorotocephala*, a representative of the free-living flatworm lineage, was undertaken.

## 2. Materials and methods

### 2.1. Chemicals

All buffers and substrates, as well as PMSF, EDTA, and BSA were obtained from Sigma-Aldrich (St Louis, Mo, USA). Auranofin was purchased from ICN Biomedicals Inc. (USA). The affinity matrix 2’5’ ADP-Sepharose 4B was obtained from Pharmacia while Trypsin was purchased to Promega (Fitchburg, MA, USA). All reagents needed for electrophoresis were obtained from BioRad. In the preparation of all buffer solutions, reverse osmosis purified water was used.

### 2.2. Biological material

Planarians belonging to the species *Dugesia dorotocephala* were collected at Cuemanco freshwater basin, Xochimilco, México City, using chicken meat as bait. The site at which planarians thrive is a public access locality and hence no official permit for either to access or to collect planarians was required. The identification of the species was carried out by using the Identification Manual Freshwater Planarians (Turbellaria) of North America [26]. A search at the International Union for Conservation of Nature (IUCN) revealed *D*. *dorotocephala* is not in the red list of threatened species. By microscopic analysis, it was found the sample was essentially homogeneous. Over 95% of the total planarians population did correspond to the above noted species. Worms were washed with dechlorinated water (three times) and stored at—20°C until use. The study was approved by the Ethics and Research Committees of the Facultad de Medicina, Universidad Nacional Autónoma de México (FMED/CI 098–2013).

### 2.3. Enzyme assays

Unless otherwise noted, all enzyme assays were performed at 25°C in 100 mM Tris-HCl buffer (pH 7.8) containing 1 mM EDTA (TE buffer) in a final volume of 0.6 mL.

#### 2.3.1. DTNB reduction assay for TrxR activity

This assay is based on the NADPH-dependent reduction of the artificial disulfide substrate DTNB [27]. The reaction mixture contained 100 μM NADPH and different concentrations of DTNB in TE buffer. An aliquot of the extract was incubated in the presence of DTNB for 3 min to obtain the base line. Then, NADPH was added and the increase in absorbance at 412 nm was followed. An extinction coefficient of 13.6 mM^-1^ cm^-1^ for DTNB was used in the calculations of enzyme activity. One unit of TrxR activity is defined as the NADPH-dependent production of 2.0 μmol of TNB^-^ per min at 25°C.

#### 2.3.2. GSSG-reductase activity

This assay is based on the NADPH-dependent reduction of the substrate GSSG, which is monitored by the decrease in absorbance at 340 nm [28]. An enzyme aliquot was incubated in TE buffer in the presence of 100 μM NADPH for 2 min to record the baseline at 340 nm; then, the reaction was started by adding GSSG at the corresponding concentration, and the NADPH oxidation was followed. The final assay volume was 0.6 mL. An extinction coefficient of 6.22 mM^-1^ cm^-1^ for NADPH was used in the calculations of enzyme activity. One unit GR activity is defined as the oxidation of 1.0 μmol of NADPH per minute at 25°C. To discriminate between the GSSG reduction rate due to TGR from that of the authentic GR activity, assays were performed either in the presence or in the absence of nanomolar concentrations of the gold compound auranofin, a selective inhibitor of selenocysteine dependent enzymes [29–31]. By subtracting the initial slope obtained in the presence of auranofin from that calculated in its absence, the GSSG reductase activity of both GR and TGR could be estimated.

#### 2.3.3. Catalase activity

The assay is based on the H_2_O_2_ oxidation, which is monitored by the decrease in absorbance at 240 nm. An enzyme aliquot was incubated in 50 mM sodium phosphate buffer (pH 7.0) for 2 min to record the baseline at 240 nm; then, reaction was started by adding H_2_O_2_ and its consumption was followed. The final assay volume was 0.6 mL. An extinction coefficient of 43.6 M^-1^ cm^-1^ for H_2_O_2_ was used in the calculations of enzyme activity. One unit of catalase activity is defined as the oxidation of 1.0 μmol of H_2_O_2_ per minute at 25°C [32].

#### 2.3.4. Effect of pH and temperature on the GR- and TGR-dependent reduction of GSSG

The pH dependence of the GR activities obtained from *D*. *dorotocephala* was determined by measuring the velocity of NADPH consumption as described above either in the absence or in the presence of 20 nM auranofin. An enzyme aliquot was incubated during 3 min in the corresponding buffer solution in the presence of 100 μM NADPH to obtain the base line; then, reaction was started by adding GSSG at a final concentration of 67 μM. In those enzyme assays performed in the presence of auranofin, a final concentration of 20 nM of the gold compound was used. The following buffer solutions, at 0.1 M final concentration were used: acetate (pH 5.3), Imidazole (pH 6.0 to 7.0), Tris-HCl (pH 7.5 to 8.2), and Tricine (pH 8.8).

The effect of temperature on the GSSG reductase activities was studied from enzyme assays carried out in the temperature range 15°C to 65°C either in the absence or in the presence of 20 nM auranofin. An enzyme aliquot was incubated during 3 min in TE buffer at the corresponding temperature in the presence of 100 μM NADPH to obtain the base line. The reaction was started by adding a small GSSG aliquot. In all cases, the pH value of the buffer solution was adjusted at the corresponding temperature.

### 2.4. Electrophoresis

Polyacrylamide gel electrophoresis under denaturing conditions was used to monitor purification of the disulfide reductase activities from *D*. *dorotocephala*. All the conditions were essentially the same as previously described [33]. Molecular weight markers were run in parallel to estimate the enzyme subunit molecular weight.

### 2.5. Protein determination

A variant of the dye-binding technique of the Lowry method was used to determine protein concentration [34]. Bovine serum albumin was used as the standard. The protein concentration of the purified preparation was determined spectrophotometrically from its FAD content by reading the absorbance at 460 nm (ε = 11.3 mM^-1^ cm^-1^). The relative abundance of the protein bands obtained after PAGE was estimated through the software IMAGEJ.

### 2.6. Tandem mass spectrometry (LC/ESI-MS/MS)

For the identification of the protein bands obtained by denaturing gel electrophoresis, the following protocol was used. Protein bands were excised from the Coomassie-stained gel, distained, and reduced with dithiothreitol (DTT). After alkylation with iodoacetamide (IAA) samples were partially digested with trypsin. Separation of the resultant peptides was performed through the methods of CID (Collision-Induced Dissociation) and HCD (High energy Collision Dissociation). Molecular mass determination of the peptides was carried out using a Hybrid Orbitrap-XL mass spectrometer from ThermoFisher Co (San Jose, CA, USA), with a nano-electro spray ionization source (ESI) [35].

### 2.7. Isolation of disulfide reductases from *D*. *dorotocephala*

#### 2.7.1. Obtention of the cytosolic fraction

Planarians were suspended in an equal volume of 20 mM Tris/HCl buffer (pH 7.2) containing 1 mM EDTA and 86 μM PMSF, and mechanically disrupted by a motor-driven Teflon pestle. The homogenate was centrifuged at 180 *g* for 20 min at 4°C, and the resultant supernatant was then centrifuged at 14600 *g* for 15 min. The pellet containing mitochondria was discarded, and the supernatant was centrifuged at 104,000 *g* for 40 min to obtain the cytosolic fraction.

#### 2.7.2. Purification of the disulfide reductases from *D*. *dorotocephala*

All purification steps were carried out at 4°C. The cytosolic fraction was dialyzed overnight against 20 mM imidazole/HCl buffer (pH 7.2) containing 1 mM EDTA and then adsorbed onto a DEAE-Cellulose column (2.6 x 20 cm) previously equilibrated in the same buffer. After washing the non-adsorbed protein, the retained proteins were eluted with a linear NaCl gradient (0–0.5 M) prepared in eight column volumes of the same imidazole buffer. Fractions containing both DTNB and GSSG reductase activities were pooled and dialyzed against 5 mM sodium phosphate buffer (pH 7.0).

The dialyzed sample was adsorbed onto a HA-Ultrogel column (2.2 x 7.0 cm) previously equilibrated in the same phosphate buffer. After washing, the column was resolved with seven column volume of a linear sodium phosphate gradient (0.005–0.5 M) at pH 7.0. Fractions containing the disulfide reductase activities were pooled and dialyzed against 10 mM Tris-HCl buffer (pH 7.8) containing 1 mM EDTA (TE buffer).

The sample was loaded onto a 2′, 5′-ADP-sepharose 4B column (2.6 x 3.0 cm) previously equilibrated in TE buffer. After washing the column with the same buffer, the enzyme was eluted by a pulse of 200 μM NADPH prepared in TE buffer (three times the column volume). Active fractions containing both disulfide reductase activities were pooled, dialyzed against the same buffer and concentrated by ultrafiltration (Amicon Ultra, Millipore, Bedford, MA, USA). The purified fraction was stored at -20°C until use.

### 2.8. Statistics

All the kinetic parameters reported in the present work were obtained from initial velocity experiments and represents the average of three independent measurements. Data are reported as mean ± standard deviation. Fitting of data to the Michaelis-menten equation was carried out through non-linear regression by using the Sigmaplot software.

## 3. Results

### 3.1. Purification and mass spectrometry analysis of the disulfide reductases from *D*. *dorotocephala*

Starting with the cytosolic fraction obtained from *D*. *dorotocephala* biomass, it was possible to isolate both GSSG reductase and Trx reductase activities. It is worth to note that through all the purification procedure, the two disulfide reductase activities were eluted in the same chromatographic peak (S1 Fig). By using GR activity to monitor the purification procedure, a yield of 40% was obtained with a purification factor of about 2700. A summary of the purification procedure is shown in Table 1.

**Table 1:** Summary of the purification procedure of the GSSG reductase activity from *Dugesia dorotocephala*.

Fraction	Vol (mL)	Protein (mg)	Specific activity (U*/mg)	Total Activity (U)	Purification (fold)	Yield (%)
Crude extract	104.0	1,160.0	0.0092	10.67	1.0	100.0
DEAE-cellulose	54.0	490.0	0.0160	7.84	1.7	73.5
HA-Ultrogel	37.0	180.0	0.0360	6.48	3.9	60.7
2,5′ADP-sepharose	0.6	0.174	24.72	4.30	2,687	40.3

* An enzyme unit is defined as the amount of protein needed to oxidize one μmol of NADPH per minute at 25°C

Date were obtained at 25°C and pH 7.8, using 67 μM GSSG as substrate.

**Fig 1:**
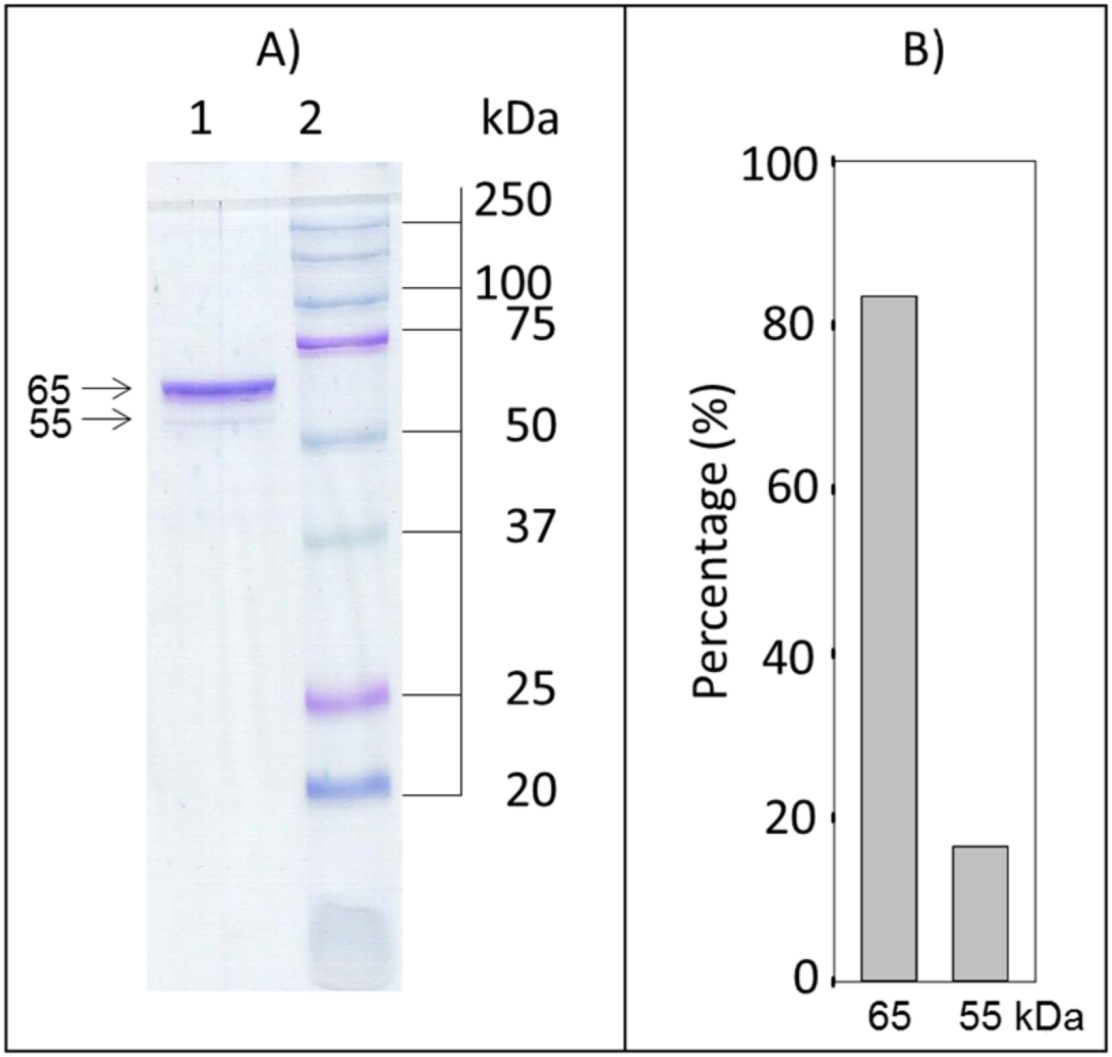
Electrophoretic analysis of the disulfide reductase activities purified from *D*. *dorotocephala*. A) Protein band pattern of the purified sample. Lane 1: An aliquot from the last purification step was incubated for 10 min at 80°C in the presence of 1% SDS and 5 mM β-mercaptoethanol; then, it was loaded on a top of a 12% polyacrylamide gel and ran during 3 h. Lane 2: molecular weight markers. B) Densitometric analysis of the 65 kDa and 55 kDa protein bands. The relative intensity of the latter was estimated with the software IMAGEJ.

The electrophoretic analysis of the purest fraction revealed the presence of two protein bands (Fig 1A). A major one located at a molecular weight of ~65 kDa representing about 85% of the total protein (Fig 1B) and a minor one of 55 kDa.

From the estimated molecular weight for the major protein band, the presence of TGR is strongly suggested. As regard the minor protein band, its molecular weight is in the expected range for either GR or TrxR. The identity of the protein bands was elucidated by mass spectrometry analysis after its tryptic digestion. From the 65 kDa protein band six different polypeptide sequences, representing a total of 92 amino acid residues, were obtained. By comparing with related sequences obtained from the Genebank data base (gb|AIC37513.1), as well as with that reported by Salinas et al (2010), a significant match with TGR from the free-living flatworms *Dugesia japonica* (81.5% global identity) and *S*. *mediterranea* (80.4% global identity) was obtained (Table 2). The sequence identity of the peptide fragments decreases slightly when compared with TGRs sequences of parasitic flatworms (data no shown). No other protein was detected in the 65 kDa electrophoretic band.

**Table 2:** Amino acid sequence of proteolytic fragments obtained from the electrophoretic bands of the purified sample of *D*. *dorotocephala*.

Protein band obtained by SDS-PAGE	Peptides sequenced obtained By LC/MS/MS	Matched peptide Sequence to
		*S*. *mediterranea* and *D*. *japonica* TGR
**~ 65 kDa**	**VE**L**NLL**QDGA**EIQ**SA**L**HTI**TNQ**K	**49–71**
	**TWGLGGTCVNVGCIPK**	**148–163**
	**DY**L**NAY**AE**FID**G	**223–234**
	**ITSDDLFSL**P**Y**C**PG**	**275–289**
	EGD**FNTVLFA**L**GR**	**378–390**
	**AGEITQGYA**V**AM**K	**541–553**
		*S*. *mediterranea* and *D*. *japonica* GR
**~ 55 kDa**	V**I**CDVD**CL**L**WA**I**G**	**259–270**
	RA**LLTPVAIAAG**	**318–329**
	R**LDY**S**NIPTV**VFSHPPI	**344–360**
	**G**TV**G**L**TE**D**EA**ISV**YG**K	**361–376**
		*S*. *mediterranea* and *D*. *japonica* TrxR
	**V**L**GGGSGGLA**ASK	**39–50**
	**WGLGGTCVNVGCIPK**	**74–90**
	**L**SG**G**LQ**LTPVA**LQ**AG**K	**369–383**
	**TPLEYA**GI**G**LA**EE**DAFAK	**408–426**

TGR, GR and TrxR of *D*. *Dorotocephala* have a coverage 15, 11, 11%, respectively, of the corresponding total amino acid sequence with total scores of 395, 252 and 262, respectively, obtained by ClustalW. Access number to GenBank data of the corresponding protein from *D*. *japonica* were as follows: TGR (gb|AIC37513.1); GR (gb|ADU86893.1); and TrxR (gb|AIC37512.1). letters in bold represent identical residues identical to that reported in both *S*. *mediterranea* and *D*. *japonica*.

As regard the ~55 kDa protein band, representing about 15% of the total protein in the purified fraction (Fig 1B), the tryptic digestion yielded eight polypeptide fragments. Four of the latter showed a significant identity percentage with GR from both *D*. *japonica* (75.8% identity) and *S*. *mediterranea* (72.4% identity). The additional four peptides matched with the amino acid sequence of TrxR from the same free-living flatworms, giving identities of 73.7% and 70.5% with the homologous protein of *D*. *japonica* and *S*. *mediterranea*, respectively (Table 2). A higher identity percentage of 81.9 was obtained when these latter peptides were aligned with the amino acid sequence of TGR from either *D*. *japonica* and *S*. *mediterranea*. This figure is essentially identical to that obtained with the peptide fragments derived from the 65 kDa protein band. Additional minor peptide fragments in the 55 kDa protein band revealed the presence of malic enzyme and catalase (data not shown).

### 3.2. Kinetic evidence for the presence of an independent GR activity in *D dorotocephala*

**Fig 2:**
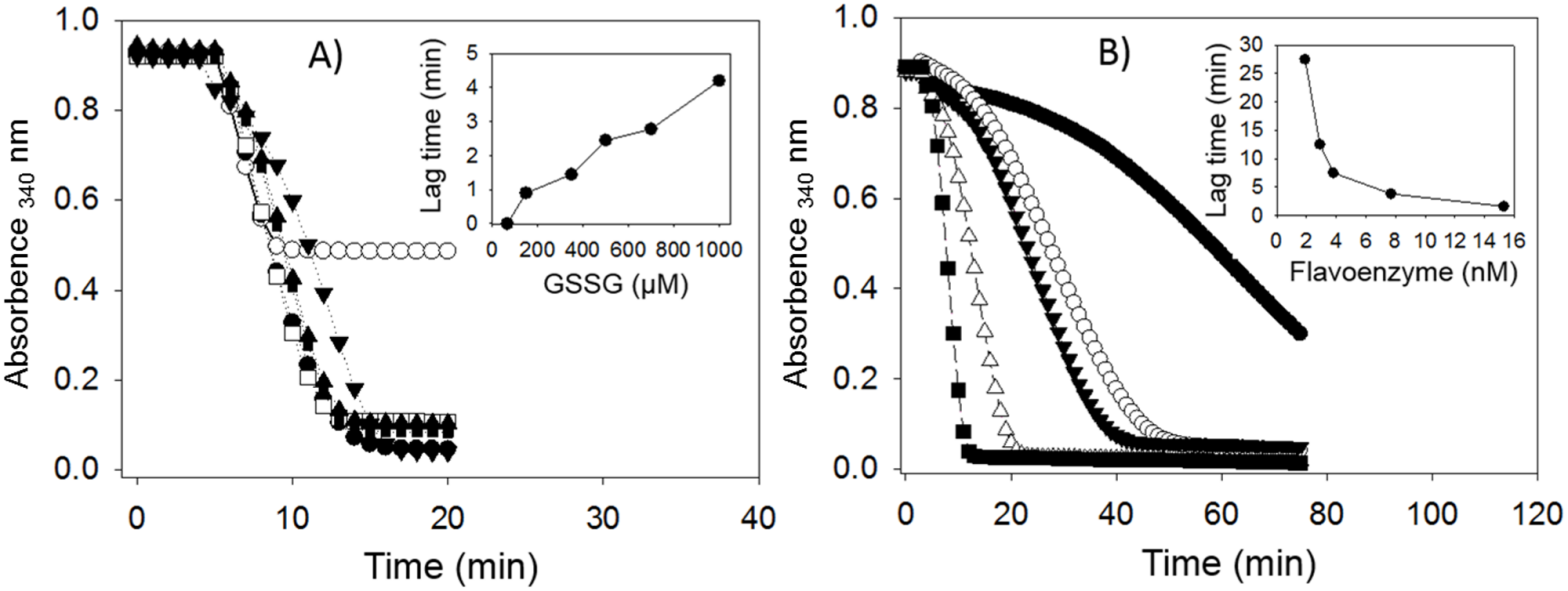
Effect of GSSG and protein concentration on the full progress curves of NADPH consumption with GSSG as substrate. The GSSG reductase activity was measured at 25°C and pH 7.8 as described in Materials and Methods. A) Effect of GSSG. The enzyme assays were carried out at the following micromolar concentrations of the disulfide: (○) 67; (□) 150; (●) 350; (■) 500; (▲) 700; (▼) 1000. An enzyme concentration of 15 nM was used. B) Effect of protein concentration. The following nanomolar concentrations of protein were used: (●) 1.9; (○) 2.9; (▼) 3.8; (Δ) 7.7; (■) 15.3. Inset at panels A and B shows the dependence of the lag time on either GSSG or protein, respectively.

A feature of TGR from parasitic flatworms is the existence of a strong substrate inhibition when GSSG is used as a substrate at concentrations above 100 μM. Such inhibition is temporary, resulting in hysteresis-like full time courses [21,23,24,36,37]. To elucidate if such phenomenon was present in the preparation obtained from *D*. *dorotocephala*, enzyme assays in the presence of increasing concentrations of GSSG were carried out. As shown in Fig 2A, an atypical progress curve was evident only at 1 mM GSSG; however, the magnitude of the lag time was barely 4 min. By decreasing the enzyme concentration in the assay mixture, a significant increase in lag time was obtained (Fig 2B). Under these conditions, it was possible to observe a lag time as great as 27 min with 1.9 nM of *D*. *dorotocephala* TGR. Such dependence of lag time on enzyme concentration is also typical of the hysteretic-like behavior of TGR [23,24,36]. Clearly, the atypical kinetic behavior of TGR from parasite flatworms is also present in the *D*. *dorotocephala* enzyme, further demonstrating such phenomenon is a common feature of TGR from any flatworm.

The low size of the lag time observed with the *D*. *dorotocephala* enzyme preparation when the GSSG concentration was varied (Fig 2A) can be explained as consequence of the concomitant presence of a conventional GR enzyme, which accompanied TGR through all the purification steps. The activity of GR will result in the production of GSH, which is known to be able to revert or prevent the hysteretic-like progress curves of TGR [21,23,24,36]. Thus, the production of GSH by GR explains the minor lag times observed in the enzyme assays with the *D*. *dorotocephala* preparation.

**Fig 3:**
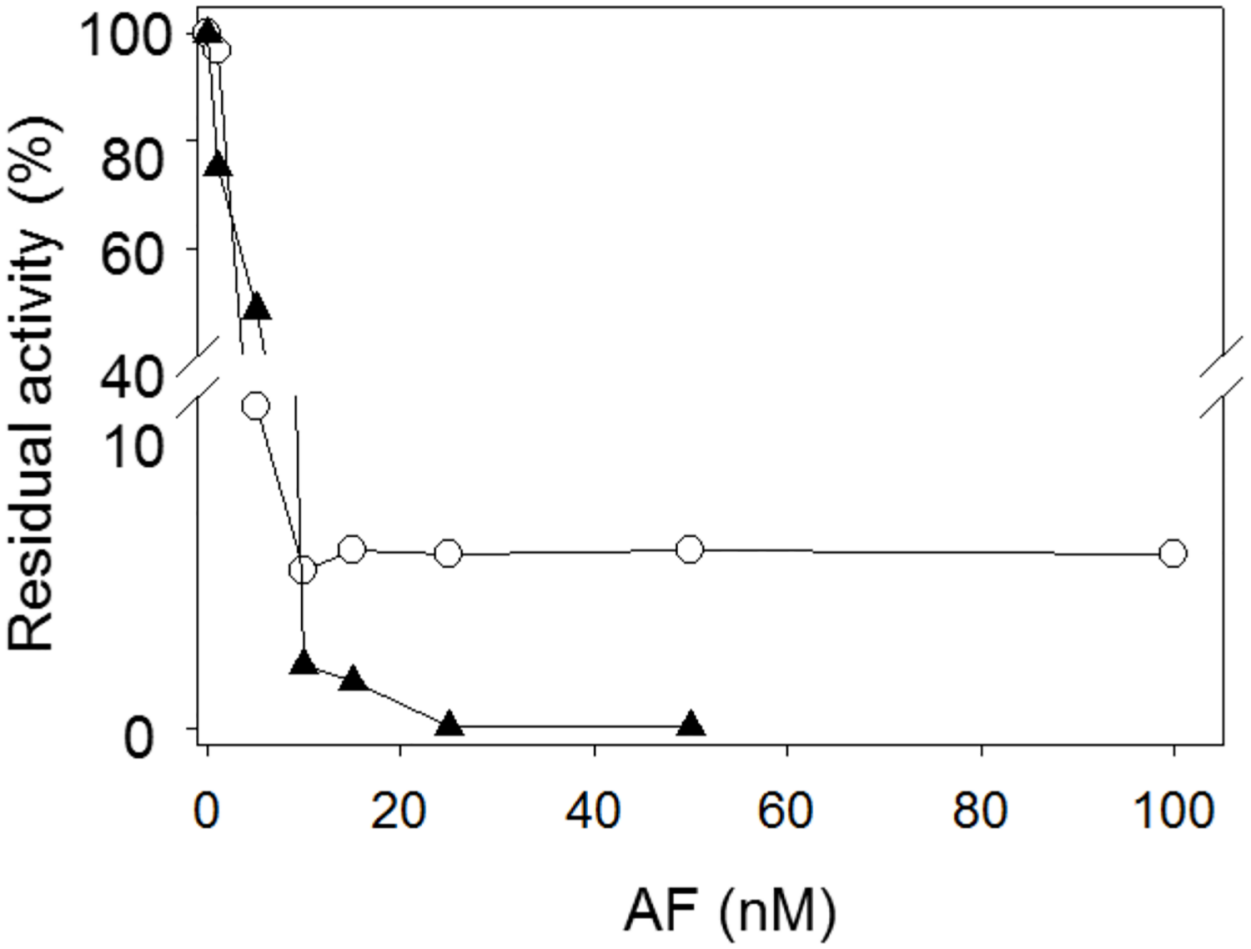
Effect of auranofin on the disulfide reductase activities of *D*. *dorotocephala*. An enzyme aliquot was incubated during 3 min in the presence of the corresponding concentration of auranofin and 100 μM NADPH; then, reaction was started by addition of either GSSG (○) or DTNB (▲) at final concentrations of 67 μM or 490 μM, respectively. The initial velocity obtained in the absence of auranofin was taken as 100% relative activity.

To discriminate between the GSSG reduction associated with TGR from that due to authentic GR, enzyme assays were performed in the presence of the gold-containing compound auranofin, an effective inhibitor of selenocysteine dependent enzymes in the nanomolar concentration range [29,30]. In this same range, auranofin has no effect on the activity of GR [30]. Fig 3 shows the dependence of the disulfide reductase activities of the purified fraction on auranofin concentration with either GSSG or DTNB as substrate. Above 15 nM of the gold compound, a non-sensitive minor GSSG reductase activity remained. Such residual activity, representing about 5% of the total GSSG reductase, can be assigned to the authentic GR found in *D*. *dorotocephala* by mass spectrometry analysis. By contrast, no residual activity was observed when the enzyme assays were performed with DTNB as the disulfide substrate (Fig 3). These results support the presence of an active and independent GR in the free-living flatworm *D*. *dorotocephala*.

**Fig 4:**
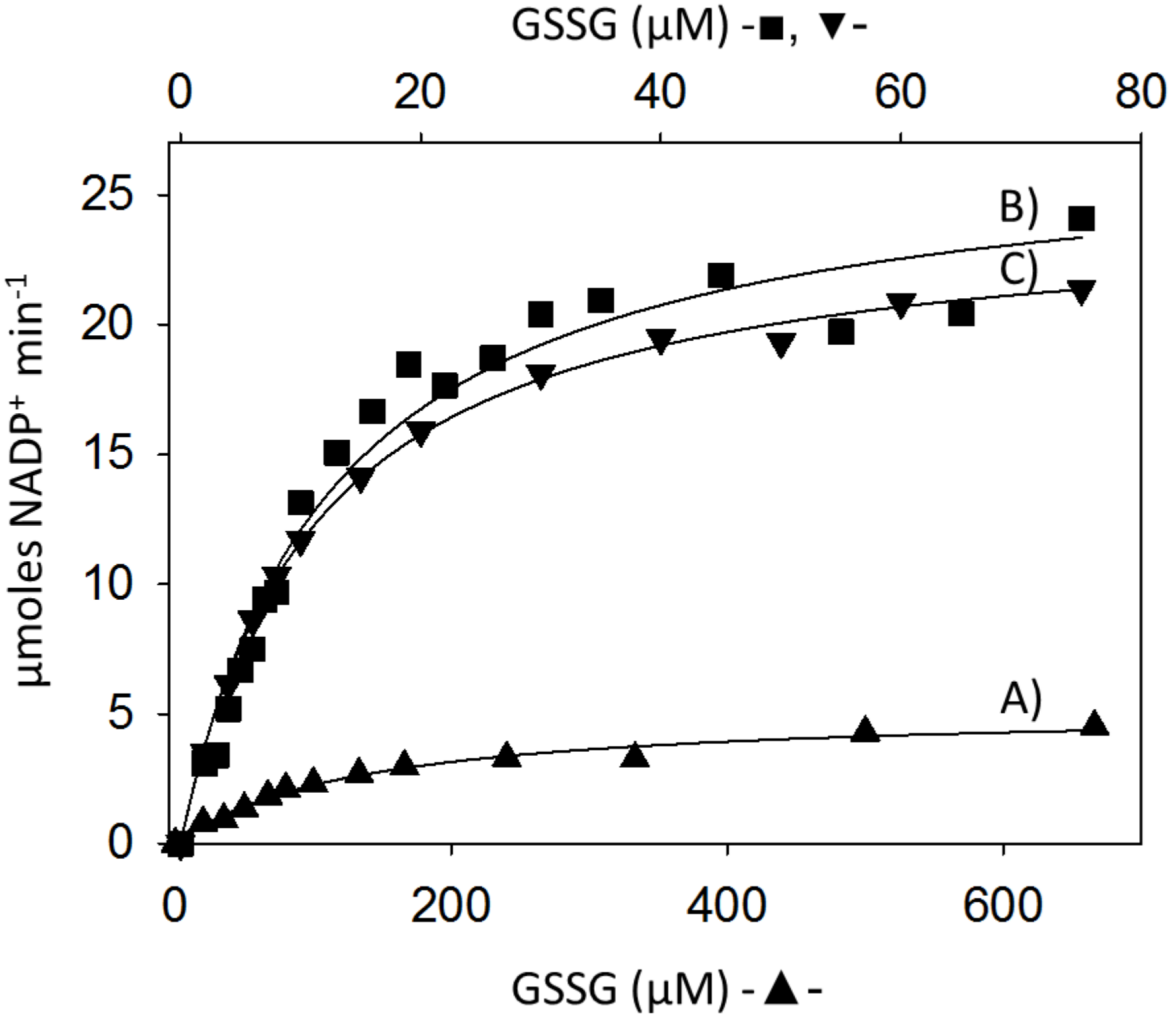
Saturation kinetics of the GR activities of *D*. *dorotocephala*. Enzyme assays were carried out as described in Materials and Methods. In all cases a NADPH concentration of 100 μM was used. (■) Total GSSG reductase activity (TGR + GR); (▲) GSSG reductase activity due to GR; (▼) GSSG reductase activity due to TGR. An enzyme concentration of 6 nM was used in the assays. The upper abscissa represents the range of GSSG concentrations used for the kinetic analysis of TGR. The continuous line was obtained through non-linear regression analysis of the corresponding data to the Michaelis-Menten equation. In all cases, error bars were omitted for clarity.

Based on the above results, steady-state kinetic experiments with GSSG as the variable substrate were carried out. Fig 4 shows the dependence of the disulfide reductase activity on GSSG concentration, either in the absence or in the presence of 20 nM auranofin. After subtracting the GR activity obtained in the presence of the gold compound from the total GR activity obtained in its absence, it was possible to obtain the true initial velocities of GSSG reduction associated with either GR or TGR from *D*. *dorotocephala*. To estimate the *K*_m_ and *V*_m_ values, the corresponding data were fitted to the Michaelis-Menten equation through a nonlinear fitting procedure. For TGR, the results were 11.5 ± 0.38 μM and 24.5 ± 0.26 μmol min^-1^ for *K*_m_ and *V*_m_, respectively, while for GR the corresponding values were 135.4 ± 8.4 μM and 5.36 ± 0.13 μmol min^-1^. Hence, under saturation conditions the contribution of the authentic GR represents about 18% of the total GSSG reductase activity. Due to the presence of contaminant enzymes in the protein band where GR was identified, no attempt to determine its specific activity was done.

**Fig 5:**
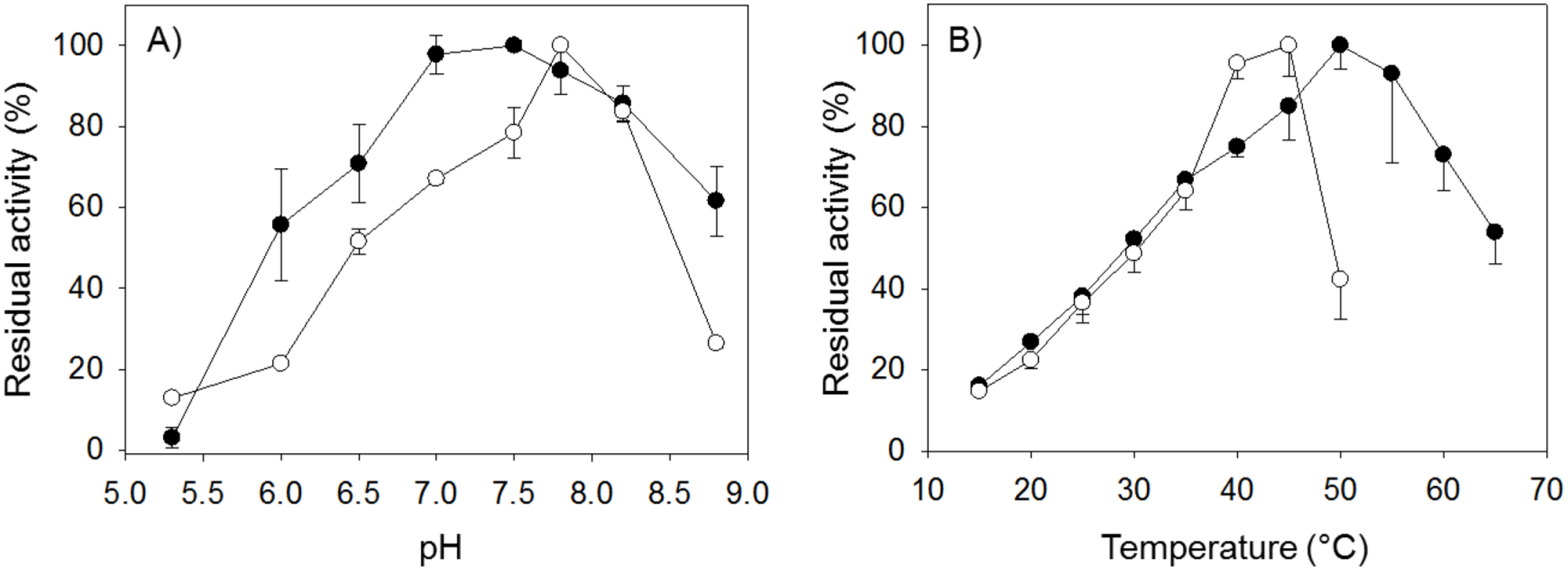
Dependence of the GSSG reductase activities of *D*. *dorotocephala* on both pH and temperature. Enzyme assays were carried out as described under Materials and Methods. The GSSG reductase activity associated with TGR (○) was obtained with 67μM GSSG. The GSSG reductase activity due to GR (●) was determined with 660 μM GSSG in the presence of 30 nM auranofin.

The presence of two independent enzyme activities in *D*. *dorotocephala* associated with GSSG reduction was further supported by determining the effect of both pH and temperature on the GR activities. As shown in Fig 5, the activity profile of GR activity on both pH and temperature was different either in the presence or in the absence of auranofin. The optimum of pH was slightly higher (Fig 5A) for the auranofin-sensitive GR activity (i.e. TGR) when compared with the corresponding value for the non-sensitive GR activity (i.e. GR). Such difference between both enzymes was further supported from the temperature dependence studies. Thus, while TGR was strongly inactivated at temperatures above 45°C, GR retained significant activity up to 65°C (Fig 5B). From the ascending branch of the curves, activation energies of 21.4 kJ mol^-1^ and 17.4 kJ mol^-1^ for TGR and GR, respectively, were calculated.

### 3.3. Evidence for the presence of an independent TrxR activity in *D dorotocephala*

**Fig 6:**
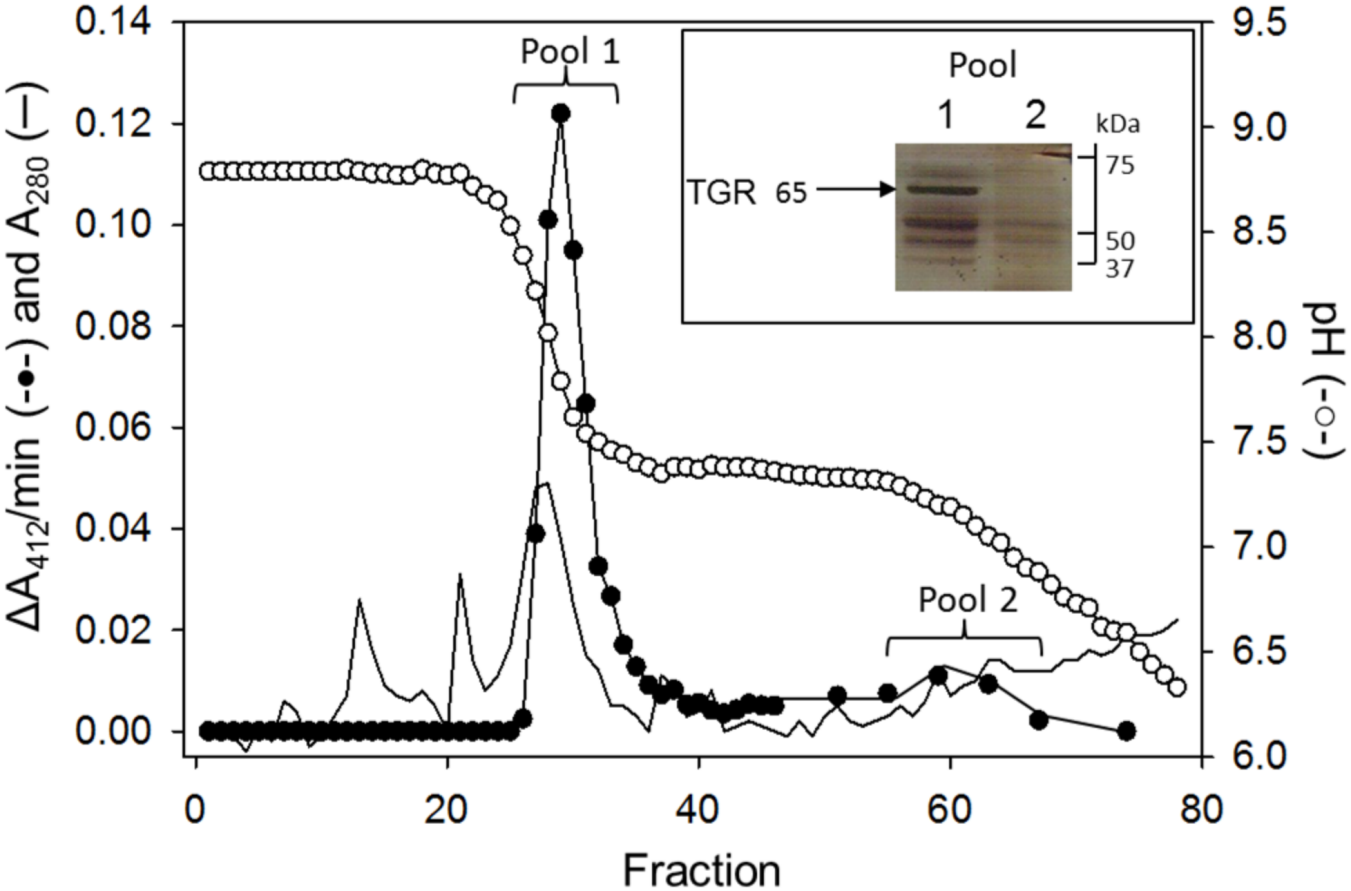
Chromatofocusing separation of the TrxR activities of *D*. *dorotocephala*. An active fraction was loaded on a DEAE-cellulose column (1.6 x 17 cm) previously equilibrated in 25 mM Tris/HCl buffer (pH 8.5). After washing, the adsorbed proteins were eluted by application of a polybuffer solution supplemented with 25 mM Hepes and 25 mM Tris (final pH of 6). Fractions containing DTNB reductase activities were pooled separately and concentrated. The major active peak (Pool 1) contain activities associated with TGR, GR and TrxR, while in the minor active peak (Pool 2) TGR is essentially absent. Insert: electrophoretic profile of both Pool 1 (line 1) and Pool 2 (line 2) after silver staining.

As shown in Fig 3, all the DTNB reductase activity present in the *D*. *dorotocephala* extract was auranofin sensitive. Thus, it was not possible to discriminate an independent TrxR activity from that due to TGR based on enzyme assays. In an attempt for to demonstrate the presence of a TGR-independent TrxR activity, a *D*. *dorotocephala* sample was subjected to chromatofocusing. The application of a pH gradient to the column made possible the separation of two peaks with DTNB reductase activity (Fig 6), which were collected separately and concentrated. Electrophoretic analysis of the two fractions (named as Pool 1 and Pool 2) revealed a different protein content. Although in both pools protein bands with the expected molecular weight for TrxR and GR was revealed, the protein band corresponding to TGR was present only in Pool 1 (Inset of Fig 6). On the other hand, enzyme assays with either DTNB or GSSG as substrates revealed the presence of both TrxR and GR activities in the two fractions although with a different activities ratio. Thus, while for Pool 1 a value of 11 for the TrxR/GR activities ratio was determined due to the presence of both GR and TGR, the corresponding value for Pool 2 was found to be 2. Additionally, the sensitivity of the GR activity to auranofin of the two fractions was also different, being higher for Pool 1. These results suggest in *D*. *dorotocephala* a TrxR is expressed as a functional protein. However, considering that TGR represents about 85% of the disulfide reductases, the contribution to the total TrxR activity must be a minor one.

## 4. Discussion

Platyhelminths represent a very old lineage of acoelomated triploblastic metazoa, which are characterized by the absence of both circulatory and respiratory systems. Although a major of the platyhelminth species have adopted a parasitic mode of life, the earlier representatives of the group arose as free-living organisms [38]. To date, a considerable number of free-living flatworm species are still alive [38]. Within the latter, the tricladids or planarians are well known, primarily due to its use as a model to study the cellular basis of regeneration [39,40]. As compared with its parasitic counterparts, the free-living flatworms have evolved under very different environmental conditions, and hence they are subjected to a wider range of oxygen tensions. Thus, it is expected they shows different physiological and/or metabolic adaptations to contend with. However, no study dealing with the enzyme complement involved in its redox metabolism is available.

Recently, genes coding for both TrxR and GR were reported in the genome of the free-living flatworm *S*. *mediterranea* [25], and it was suggested that its presence is related to the life style of this organism. Nevertheless, the presence of a specific gene does not probe the occurrence of the protein, the experimental demonstration of its expression is needed. In this work we have been able to copurify the three disulfide reductases GR, TrxR and TGR from the adult stage of the free-living flatworm *D*. *dorotocephala*. The presence of independent GSSG reductase activities due to GR and TGR was clearly demonstrated by mass spectrometry analysis of peptide fragments obtained from the purified fraction, as well as from kinetic experiments. The use of the gold compound auranofin as a selective inhibitor of selenium dependent thioredoxin reductases did allow us both discriminate between the GSSG reductase activity due to TGR from that produced by GR, as well as to obtain its kinetic parameters. Nanomolar concentrations of auranofin were high enough to fully inhibit the disulfide reductase activities associated with TGR. Under such conditions, it was found that barely 5% of the total GR activity can be assigned to GR, strongly suggesting TGR is the main enzyme involved in the reduction of GSSG in *D*. *dorotocephala*. Hence, it can be concluded that in *D*. *dorotocephala* genes coding for both GR and TGR are translated into functional proteins. However, although in a free-living platyhelminth a functional GR is expressed, TGR remains as the major disulfide reductase involved in GSSG reduction.

In spite its low contribution to the total GSSG reductase activity, GR could play an important physiological role in some tissue of *D*. *dorotocephala*. It must be noted that in *Fasciola hepatica* TGR is located mainly to the male reproductive system, in addition to the parenchyma [41]. In this sense, a specialized role for TGR in mouse testes was proposed, being involved in spermatid maturation [19]. Thus, it is possible that in free-living platyhelminths, in which both TGR and GR are present as functional enzymes, GR could plays an essential role in tissues other than the male gonads. Alternatively, the low amount of GR present in the adult stage of *D*. *dorotocephala* could represent vestigial expression of two enzymes which were important in earlier stages of development. More work is needed to clarify this issue.

A comparison of the kinetic parameters of both GR and TGR from *D*. *dorotocephala* with that of the homologous enzymes from a diversity of sources is shown in Table 3. For GR, the *V*_m_ and *K*_m_ values are in the range reported for typical GR. The specificity constant (i.e. the *V*_m_/*K*_m_ ratio) is also in the expected range (~ 10^6^ M^-1^ s^-1^). As regard *D*. *dorotocephala* TGR, the *K*_m_ (11 μM) determined in the present work is in the lower range as compared with the corresponding value available for the enzyme from parasite flatworms, while the *k*_cat_ (26.5 s^-1^) is the highest so far reported for any TGR. Taken altogether, these values yield a specificity constant value of 2.4 x 10^6^ s^-1^ M^-1^ for *D*. *dorotocephala* TGR, which is about one order of magnitude higher than the corresponding value of the homologous enzyme from other sources. Interestingly, such value is in the range of that of authentic GR. Unfortunately, no information is yet available for TGR from any other free-living flatworm.

**Table 3:** Kinetic parameters of GR and TGR from *D*. *dorotocephala* compared with the corresponding values from other sources.

Reductases	*K*_m_ (μM)	*k*_cat_ (s^-1^)	*k*_cat_/*K*_m_ (M^-1^s^-1^)	References
^a^***Sc*_GR**	590.0	535.0	0.9 x 10^6^	[46]
^b^***Rn*_GR**	53.1	258.0	4.8 x 10^6^	[47]
^c^***Hs*_GR**	72.0	165.0	2.3 x 10^6^	[48]
^d^***Pf*_GR**	95.0	120.0	1.3 x 10^6^	[48]
^e^***Bt*_GR**	42.0	175.0	4.2 x 10^6^	[49]
^f^***Dd*_GR**	136.2	125.0	0.9 x 10^6^	[This Work]
^g^***Mm*_TGR**	8.8	1.6	1.7 x 10^5^	[18]
^h^***Sm*_TGR**	71.0	21.0	3.0 x 10^5^	[50]
^i^***Sj*_TGR**	49.5	5.4	1.1 x 10^5^	[50]
^j^***Me*_TGR**	76.0	5.3	0.7 x 10^5^	[51]
^k^***Tc*_TGR**	15.0	5.1	3.4 x 10^5^	[24]
^m^***Dd*_TGR**	11.1	26.5	2.4 x 10^6^	[This work]

The particular conditions of pH and temperature at which the GSSG reductase activity was determined, as well as the meaning of the abbreviatures is as follows:

^a^*Sc*: *Setaria cervi* pH 7.0 at 37°C;

^b^*Rn*: *Rattus norvegicus* pH 7.4 at 37°C;

^c^*Hs*: *Homo sapiens*:

^d^*Pf*: *Plasmodium falciparum* pH 7.4 at 25°C;

^e^*Bt*: *Bos Taurus* pH 7.0 at 30°C;

^f^*Dd*: *Dugesia dorotocephala* pH 7.8 at 25°C;

^g^*Mm*: *Mus musculus* pH 7.5 at 25°C;

^h^*Sm*: *Schistosoma mansoni*;

^i^*Sj*: *Schistosoma japonicum* pH 7.4 at 25°C;

^j^*Me*: *Moniezia expansa* pH 7.2 at 37°C;

^k^*Tc*: *Taenia crassiceps* pH 7.8 at 25°C;

^m^*Dd*: *Dugesia dorotocephala* pH 7.8 at 25°C

The dependence of the GSSG reductase activities from *D*. *dorotocephala* on pH and temperature revealed additional differences between GR and TGR. Thus, the maxima of activity of the latter was located about pH 8, while for GR the optimal pH was found to be at 7–7.5. Furthermore, the non-overlapping pH dependence profiles suggests catalytic groups with different p*K*_a_ values are involved in GSSG reduction by GR or TGR. As to the effect of temperature, it is worth to note the higher sensitivity of the GSSG reductase activity of TGR at the higher temperatures tested as compared with GR. Thus, while above 45°C TGR is readily inactivated, GR retains significant activity up to 65°C (Fig 5).

On the other hand, the results of the present work revealed TGR from *D*. *dorotocephala* also displays the peculiar inhibitory effect with GSSG at moderate or high concentrations (Fig 2), like the enzyme from its parasitic counterparts [21,23,24,36,37]. This fact strongly suggests such kinetic behavior is a common feature of TGR from any flatworm. Although the molecular origin of the hysteretic-like kinetic behavior of TGR has not been elucidated, it has been proposed that the temporary inhibition of the GR activity of TGR by GSSG could act as a mechanism to protect protein thiols from oxidation during an oxidative event through reversible glutathiolation [21]. Considering that the atypical substrate inhibition of TGR depends on the concentration of GSSG, GSH and enzyme, the values of such parameters in an organism will be critical for to determine the magnitude of such phenomenon. Thus, a combination of a low GSH/GSSG concentration ratio concomitant with a low concentration of the enzyme *in vivo*, could potentially produce the temporary inhibition.

As regard TrxR, its presence as an independent functional enzyme in *D*. *dorotocephala* is suggested by two experimental findings. First, the obtention of two fractions with DTNB reductase activity from the chromatofocusing experiment (Fig 6), one of which is essentially free of TGR. Second, the pattern of proteolytic digestion of the protein bands separated by electrophoresis, such that the amino acid sequence of the fragments with identity to TrxR from the 55 kDa protein band is clearly different from that obtained from the 65 kDa protein band, thus excluding the possibility that the former could be the result of the major module of TGR in which the Grx domain to be absent.

Based on the results of the present work, it can be concluded that in the adult stage of a free-living flatworm, the status of the disulfide reductases is not very different from that found in the parasitic representatives of the group (i.e. flukes and cestodes). Thus, although the genetic information coding for GR TrxR and TGR is present in the free-living species of the flatworms, and the corresponding enzymes expressed as functional proteins, TGR is still the predominant disulfide reductase. This finding does not support the recent proposal suggesting in parasite flatworms the merging of Grx into the animal TrxR module to give TGR represented an adaptation to parasitism [42]. The results of the present work suggest that in the common ancestor of the flatworm lineage, genes coding for the three disulfide reductases GR, TrxR and TGR were already present, and that during the divergence toward the parasitic lineages only the genetic information coding for TGR was retained. In this sense, it has been proposed that during the evolution of eukaryotic genomes, gene loss is a general tendency [43]. In flatworms, such tendency is well documented [44,45]. It is worth to note that a preliminary analysis of TGR-like sequences found in the data base revealed the enzyme is present in animals as earlier as sponges and coelenterates (unpublished observations), thus refuting the proposal that TGR arose as an adaptation to parasitism.

Finally, a collateral finding of the present work was the detection of proteolytic fragments with a high identity to catalase. The fragments were obtained by electrophoresis from the minor protein band in which both GR and TrxR were found. By monitoring simultaneously catalase activity during the purification of the disulfide reductases, it becomes clear the major fraction of catalase was located at the non-adsorbed material from the affinity chromatography. Using this partially purified extract, a preliminary kinetic characterization of catalase was performed. From the resultant hyperbolic saturation curve, a specific activity of 2.87 ± 0.23 μmol mg^-1^ min^-1^ and a of *K*_m_ = 19.9 ± 3.7 mM were obtained. Hence, it can be concluded that in the adult stage of *D*. *dorotocephala* a complete set of antioxidant enzymes are present, thus enabling the free-living flatworm to contend with the oxidative stress. Such situation is in clear contrast with that found in the parasite relatives, in which only TGR is coded in the genome [20].

## 5. Conclusions

The results of the present work revealed in the adult stage of the free-living flatworm *D*. *dorotocephala* the three disulfide reductases, glutathione reductase (GR), thioredoxin reductase (TrxR), and thioredoxin-glutathione reductase (TGR) are expressed as functional enzymes However, both in its expression level and in reductase activities, TGR is the main disulfide reductase, accounting for over 90% of the total GR and TrxR activities. These results strongly suggest the merging of the Grx and TrxR modules into TGR does not arose as an adaptation to a parasite mode of life.

## Supporting information

S1 Fig Chromatographic profiles obtained during the purification of the disulfide reductase activities from the *D*. *dorotocephala* extract. A) DEAE-Cellulose ion exchange chromatography; B) Hydroxyapatite chromatography; C) 2’ 5’-ADP Sepharose affinity chromatography.
(PDF)

## Abbreviations

DTNB: 5,5′-dithiobis (2-nitrobenzoic) acid
GR: glutathione reductase
grx: glutaredoxin
GSSG: disulfide form of glutathione
TGR: thioredoxin-glutathione reductase
trx: thioredoxin
TrxR: thioredoxin reductase
