# Supplementary material for: Differential expression of disulfide reductase enzymes in a free-living platyhelminth (Dugesia dorotocephala)
Source: PLoS One. 2017 Aug 7;12(8):e0182499. doi: 10.1371/journal.pone.0182499 (PMC5546602; doi:10.1371/journal.pone.0182499)
Supplement: S1 Fig — A) DEAE-Cellulose ion exchange chromatography; B) Hydroxyapatite chromatography; C) 2’ 5’-ADP Sepharose affinity chromatography. (PDF) [file pone.0182499.s001.pdf]

S1_Figure. Chromatographic profiles obtained during the purification of the disulfide reductase activities from the *D. dorotocephala* extract.

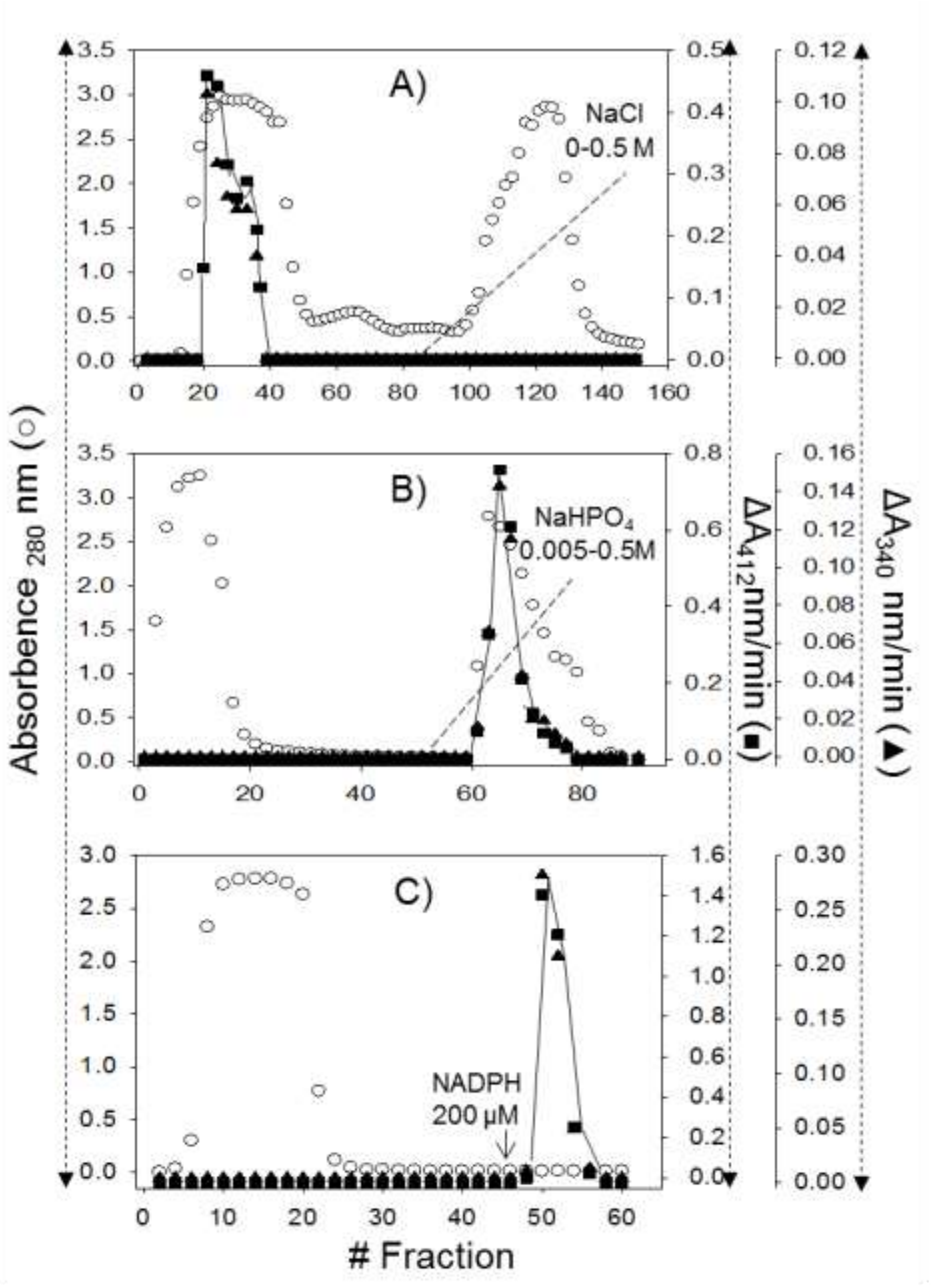

A) DEAE-Cellulose ion exchange chromatography; B) Hydroxyapatite chromatography; C) 2’ 5’-ADP Sepharose affinity chromatography.
